# Effectiveness of a Cognitive Stimulation Program in Older Adults with Mild Neurocognitive Disorder: Insights from fNIRS Analysis in a Randomized Controlled Trial

**DOI:** 10.3390/neurolint17070108

**Published:** 2025-07-15

**Authors:** Susana I. Justo-Henriques, Rosa C. G. Silva, Janessa O. Carvalho, João L. A. Apóstolo, Débora Nogueira, Telmo A. S. Pereira

**Affiliations:** 1 Department of Education, Social and Behavioral Sciences, Polytechnic University of Beja, 7800-295 Beja, Portugal; 2 Health Sciences Research Unit: Nursing (UICISA: E), Nursing School of Coimbra, 3004-011 Coimbra, Portugal; apostolo@esenfc.pt; 3 RISE-Health, Nursing School of Porto (ESEP), 4200-319 Porto, Portugal; rosasilva@esenf.pt; 4 Department of Psychology, Bridgewater State University, Bridgewater, MA 02325, USA; janessa.carvalho@bridgew.edu; 5 Coimbra Health School, Polytechnic University of Coimbra, Rua da Misericórdia, Lagar dos Cortiços, S. Martinho do Bispo, 3045-093 Coimbra, Portugal; deboramsnogueira01@gmail.com (D.N.); telmo@estescoimbra.pt (T.A.S.P.); 6 H&TRC—Health & Technology Research Center, Coimbra Health School, Polytechnic University of Coimbra, Rua 5 de Outubro, 3045-043 Coimbra, Portugal

**Keywords:** cognitive function, cognitive stimulation therapy, depressive symptoms, executive function, fNIRS, mild cognitive impairment, non-pharmacological interventions, prefrontal cortex, older adults

## Abstract

Background/Objectives: Neurocognitive disorders (NCDs) encompass a spectrum of conditions that significantly impact cognitive domains, including attention, memory, and language. Mild NCD, increasingly prevalent with aging, represents an early stage of these disorders, characterized by cognitive deficits that do not interfere with daily functioning. Non-pharmacological therapies, especially cognitive stimulation, are widely recommended to preserve cognitive function of older adults. This study aimed to evaluate the effectiveness of a 12-week individual cognitive stimulation (iCS) program on cognitive performance, mood, and prefrontal cortex activation in older adults with mild NCD using a single-blind, randomized, parallel two-arm RCT. Methods: A sample of 36 older adults were selected from a central region of Portugal. The intervention group (*n* = 18) received 24 iCS sessions, twice weekly for 12 weeks. The control group (*n* = 18) completed their regularly scheduled activities. Outcomes included global cognitive function, executive functioning, and mood. All participants were assessed at baseline and after the intervention. Functional near infra-red spectroscopy (fNIRS) was also collected to measure prefrontal cortex activity at both time points in the intervention group. Results: The intervention group showed a significant improvement in global cognition and executive functions, and reduced depressive symptomatology compared to the control group. fNIRS data revealed enhanced activation and functional efficiency in the lateral prefrontal cortex following the iCS program. Adherence and degree of collaboration to the intervention were very high. Conclusions: These findings suggest that iCS is an effective approach to improving cognitive function and mood in mildly cognitively impaired older adults.

## 1. Introduction

Global population aging is growing at an unprecedented rate [1,2]. In turn, the SARS-CoV-2 pandemic increased health vulnerabilities in older adults by reducing access to health care, driving social isolation, and increasing mood and anxiety disorders; together, this potentially has promoted the development and exacerbation of significant neurodegenerative diseases, such as mild and major neurocognitive disorders (NCDs) [3,4]. Mild NCD is an intermediate stage between healthy aging and major NCD, characterized by a modest impairment in cognitive performance, but intact independence in activities of daily living (ADLs) [5]. Major NCD is characterized by neurocognitive changes in domains such as complex attention, executive function, learning, memory, language, perceptual-motor, and social cognition. NCD has different etiological subtypes, including Alzheimer’s disease (AD), frontotemporal lobar degeneration, Lewy body disease, and vascular disease, among others [5]. Neuropsychiatric symptoms (NPSs), including agitation, aggressive behavior, apathy, psychosis, hallucinations, and disinhibition, are common yet challenging consequences of NCD [6,7]. NPSs are associated with poor outcomes, including decreased quality of life and well-being of older adults [6,7]. In 2020, over 50 million people worldwide were living with NCDs. By 2050, this number is projected to reach 152 million [8].

Considering the growing number of individuals with NCDs, efforts have focused on developing a better understanding of the presentation and management of these neurodegenerative conditions. This includes evaluating the effectiveness of various therapeutic approaches, both pharmacological and non-pharmacological, in managing NPS, enhancing cognitive function, and supporting ADLs [9,10].

Studies using different diagnostic tools such as single-photon emission computed tomography (SPECT), positron emission tomography (PET), and functional magnetic resonance imaging (fMRI), have consistently shown that the participants with NCD present reduced cerebral blood flow compared to individuals with normal cognitive function [11].

The prefrontal cortex (PFC) plays a central role in higher-order cognitive processes such as decision-making, problem-solving, planning, language, social behavior, and memory [12,13]. In individuals with neurocognitive disorders (NCDs), particularly those with Alzheimer’s disease or amnestic mild NCD, this region often exhibits hypoperfusion and reduced oxygenation during tasks [11,14]. These alterations have been linked to poorer executive functioning and other cognitive deficits when compared to cognitively healthy older adults [14]. Given its central role and vulnerability, the PFC has become a key target for evaluating the effectiveness of cognitive interventions using tools like functional near-infrared spectroscopy (fNIRS). fNIRS is a portable tool that measures tissue oxygenation in different brain regions [15]. Non-pharmacological interventions such as cognitive stimulation, training, and rehabilitation, seek to preserve cognitive, functional, and NPS abilities. While there are some criticisms about the methodology surrounding most non-pharmacological intervention studies (e.g., neurobiological mechanisms, variability among group activities), they do show gains in these areas among participants [16,17,18,19].

Cognitive stimulation (CS) is the most studied non-pharmacological intervention. This intervention can be carried out in a group or individually and focuses on intellectual and social stimulation by using relevant and engaging social and cognitive activities [18,19]. Examples of CS activities include memory exercises (e.g., recalling past events or lists), problem-solving tasks, categorization activities, word and number puzzles, creative storytelling, and discussions of current events. Additionally, sensory stimulation activities, music therapy, and guided imagery are frequently incorporated to enhance cognitive and emotional engagement [20,21,22].

This study specifically focuses on individual cognitive stimulation (iCS), which differs from group cognitive stimulation (GCS) in several key aspects. GCS typically involves multiple participants engaging in structured cognitive activities in a shared setting, fostering social interaction and peer learning. In contrast, iCS provides a one-on-one approach, allowing for personalized task difficulty adjustments and a greater focus on individual cognitive deficits [21,22,23]. Research suggests that while GCS enhances socialization and general cognitive function, iCS may be more effective in targeting specific cognitive weaknesses, ensuring higher engagement, and minimizing external distractions [21,22,23]. Moreover, iCS is often more feasible for individuals with mobility limitations or severe sensory impairments, as it can be adapted to various settings, including home-based interventions [21,22,23]. However, while we have explored the effectiveness of this individual CS (iCS) program in many samples and formats (e.g., in-home, in supervised residential settings, day programs; persons with psychiatric and various neurodegenerative disorders), it is necessary to use more reliable procedures to measure the impact of CS. One strategy may include exploring biophysiological outcomes [18], including fNIRS.

Thus, the aim of this study was to evaluate the effectiveness of an iCS program in older adults with mild NCD. Specifically, the study tested the effectiveness of iCS program on the global cognitive abilities, including executive functions, mood, and PFC activation. We hypothesized that hemodynamic adaptations in the PFC, assessed by fNIRS following iCS program constitute a physiological and psychometric indicator of improved cognitive efficiency in older adults with mild NCD.

## 2. Materials and Methods

This study reported the results of a clinical trial of iCS for older adults with mild NCD (clinicaltrials.gov ID: NCT04693611). The study was designed as a single-blind, randomized, parallel two-arm (iCS vs. treatment as usual, 1:1 ratio), controlled trial. Participants in the intervention group received 12 weekly sessions of iCS in addition to their regularly scheduled activities at the facility. Participants in the non-equivalent control group only underwent their usual activities. Participants were assessed at baseline (T0), and upon completion of the 12-week intervention (endpoint; T1). This study follows the Consolidated Standards of Reporting Trials (CONSORT) statement guidelines, and the extension for randomized trials of non-pharmacologic treatments [24,25].

### 2.1. Participants

**Figure 1:**
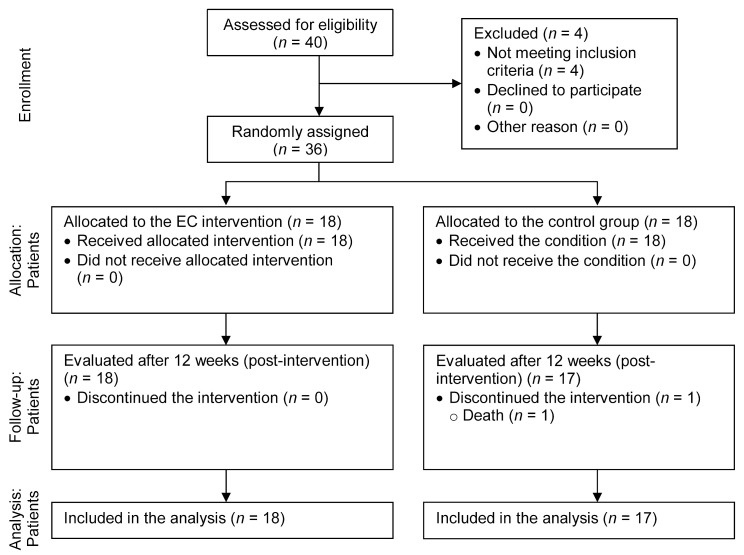
CONSORT diagram of participant flow through the study.

The CONSORT diagram shown in Figure 1 details the flow of participants recruited to the study and reasons for ineligibility. The participants were recruited from the community of a region of central Portugal. Forty participants met eligibility and inclusion criteria. After the baseline assessment, 36 participants were selected for the iCS: 18 participants were allocated to the intervention group and 18 participants to the control group.

The inclusion criteria for participants were as follows: persons with minimum age of 65 years; ability to communicate and understand written and verbal communication; native Portuguese speakers; education level of 4 years or more; Mini-Mental State Examination (MMSE) [26,27,28] score of 23 or better. The exclusion criteria for participants were as follows: suffering from an acute or serious illness, or severe sensory and physical limitations, or presence of severe neuropsychiatric symptoms that interfere with participation in interventions; history of seizures or cerebrovascular disease; diagnosis of movement disorders.

Participants who met the inclusion criteria were enrolled, and baseline assessments were completed. Participants were then randomly assigned to either the control group or the intervention group at a 1:1 ratio. Randomization was conducted through a non-stratified permuted block randomization process (with a variable block size), which was carried out using the DatInf^®^ RandList software (version 1.5, DatInf GmbH, Tübingen, Germany) to ensure allocation concealment and minimize bias. Group allocation was unknown to participants and the therapists until the intervention started. The evaluator, an external and independent professional, was not informed about group allocation and did not participate in the intervention sessions. Blinding was maintained by assigning evaluations to clinical psychologist trained specifically for this purpose. All assessments were conducted in a controlled environment at T0 and T1. Participants were evaluated individually in a quiet room to ensure consistency. The evaluator conducted the assessments after receiving standardized training in test administration. All iCS sessions were conducted by therapists (two other clinical psychologists), with more than 5 years’ experience in cognitive stimulation who also received a 7 h training on the program and principles of cognitive therapy, as well as the use of the fNIRS system by two principal investigators. Written informed consent was obtained from all participants or their legally authorized representatives prior to the intervention.

The study was approved by the ethics committee of the Health Sciences Research Unit: Nursing of Nursing School of Coimbra (number 735/12-2020). The participants contacted by the research team were informed about the study’s objectives and methods and were assured about the voluntary nature of their participation. All participants were informed about the freedom to withdraw from the study at any time. Therapists monitored participants for disinterest and offered the option to withdraw.

### 2.2. Intervention

This study was conducted in institutional care settings, ensuring controlled conditions for the intervention. The intervention consisted of a well-established iCS program [20,21,22], taking place biweekly for 12 weeks, totaling 24 sessions. The current iCS program is detailed elsewhere [20]. Each session had an average duration of 45 min, and was structured as follows: (i) welcome (greeting to the participant—5 min); (ii) orientation to time and place (10 min); (iii) cognitive stimulation main activity (25 min); (iv) process, review, and end of the session (5 min) (Table 1). The iCS sessions were individual (one-to-one) and led by a trained therapist. The sessions included several activities based on the principles of iCS and were individualized (the degree of difficulty of the exercises in the program was adjusted according to the degree of cognitive impairment). There were no repetitions of activities (i.e., each one of the 24 sessions was different, though the structure of 4 series of 6 thematic sessions was consistent) and throughout the program, the degree of difficulty was increased from session to session. Due to the SARS-CoV-2 pandemic, the program was adapted and converted into digital format (Microsoft PowerPoint presentation). Overall, the program addressed multiple cognitive domains (e.g., attention, memory, language, praxis, calculus, executive functions, gnosis) and activities were based on the therapeutic tools of CS: Roletas da Memória^©^ [Memory Roulettes], which comprised Portuguese language, math, and daily living activities, and Bingos Seniores^©^ [Senior Bingos], which includes fruit bingo, travel to the past bingo, and sound bingo. Participants were asked to remain still during the sessions due to the fNIRS equipment, and encouraged to be actively engaged in the intervention. Control group participants underwent their usual activities (without additional structured interventions). These usual activities included social interaction, physical activities, stimulation of personal skills (with no restrictions on activities, as long as they are distinct from the intervention program), and medication administration. Since those in the intervention group also additionally participated in all of their usual activities, we can presume the trial examines the additional effects of iCS. Therapists completed an adherence questionnaire after each session and were encouraged to contact the study’s principal researchers if they had any concerns about the intervention program.

### 2.3. Instruments

All participants were evaluated at T0 and T1, using a single-blind format. At T0, a structured questionnaire was administered to collect participants’ sociodemographic data, including direct questions about age, gender, educational level, marital status, and relevant medical history. During each individual cognitive stimulation session, the behavioral status of the participants was evaluated. Several tests were administered to all participants at T0 and T1, including neurocognitive tests and fNIRS recording to assess hemodynamic responses in the PFC. The fNIRS data were collected during the neurocognitive intervention at T0 and T1, following the procedure described in the next section. This decision to collect fNIRS data only at the first (T0) and last (T1) sessions was based on feasibility and methodological factors. Given the logistical constraints and the need to reduce participant fatigue in this older population, repeated fNIRS recordings across all 24 sessions were deemed impractical. Furthermore, T0 and T1 were selected as representative and clinically relevant timepoints to capture pre- and post-intervention changes in hemodynamic activity. Additionally, the subtle differences expected between each time point were not likely to be statistically or clinically meaningful and likely would only increase the risk for spurious measurement error if examined. For behavioral and neurocognitive assessments, the following instruments (Portuguese versions) were used:

The MMSE (Cronbach’s alpha = 0.89) assessed global cognitive function. Scores range from 0 to 30, with higher scores indicating better cognitive functioning [26,27,28].

The Frontal Assessment Battery (FAB; Cronbach’s alpha = 0.83) evaluated executive function on several subtests: conceptualization, mental flexibility, motor programming, sensitivity to interference, inhibitory control, and environmental autonomy. Scores range from 0 to 18, with higher scores indicating better executive functioning [29,30].

The Geriatric Depression Scale-15 (GDS-15; Cronbach’s alpha = 0.83) measured mood. It is considered a reliable self-report tool to screen depressive symptoms in older adults, in a dichotomous format (yes/no answers). Scores range from 0 to 15, with higher scores indicating more severe depressive symptoms [31,32,33,34].

### 2.4. Functional Study of the Prefrontal Cortex with fNIRS

The functional study of the PFC was conducted using fNIRS at T0 and T1, during the neurocognitive intervention, and only in the intervention group. We used only the data from the first (main activity related to the Portuguese language) and last (main activity related to sound bingo) sessions for comparison. The obtained results were significant, at baseline and after the intervention; as the data from the in-between sessions was not analyzed, we cannot speak to results of patient progress during the sessions. Each participant was seated comfortably in a room with controlled lighting, temperature, and humidity conditions, where the tasks outlined in the intervention program were implemented. During the intervention, PFC activity was monitored using a 16-channel fNIRS system (fNIR100A-2, Biopac System Inc., Goleta, CA, USA), fitted to the forehead and isolated with a dark, light-proof band. Participants were instructed to minimize head movements to reduce noise within the data. fNIRS data acquisition was performed on a dedicated computer using Cognitive Optical Brain Imaging (COBI) Studio software, version 1.5.0.24 [35], capturing real-time values of HBO and HBR variations. All results were stored digitally.

The fNIRS data were visually inspected, with low-quality channels and motion artifacts removed. Given the high susceptibility to movement-related artifacts in populations with cognitive impairment, particular attention was paid to minimizing noise during data acquisition. The raw files were then filtered using a 20th-order FIR low-pass filter with a frequency band of 0.02–0.40 Hz and a cutoff frequency of 0.1 Hz to eliminate long-term drifts, high-frequency noise, and cardiac and respiratory cycle effects. In addition to visual inspection, a validated sliding-window motion artifact rejection algorithm was applied to detect and correct transient signal disruptions, reducing the influence of non-neural signal components [36,37].

Changes in HbO2 (ΔHbO2) and HHb (ΔHHb) concentrations were calculated based on the modified Beer–Lambert law, using a 10 s baseline recorded at the start of each task. Blood oxygenation (ΔOxy) was determined by the difference between ΔHbO2 and ΔHHb, while blood volume changes (ΔHbT) were calculated by their sum [12,36,37]. All pre-processing steps were implemented in accordance with best practices for fNIRS analysis in clinical and aging populations [38].

**Figure 2:**
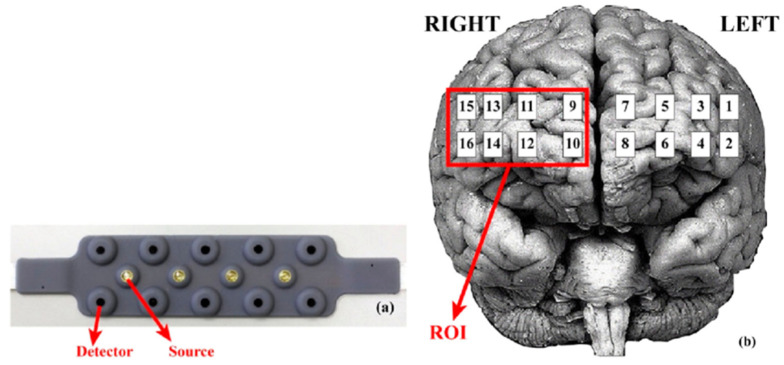
Representation of the fNIRS acquisition pad with 10 detectors and 5 LED source emitters (**a**) and the corresponding acquisition channels (16) projected over the pre-frontal cortex in relation to each brain hemisphere (**b**).

All data processing procedures were performed using fNIR software, version 4.5 (Biopac System Inc., Goleta, CA, USA). Absolute values obtained after processing were normalized (Z-scored) and outliers excluded. Data were then grouped by channel for each condition, with four regions of interest (ROI) for the PFC defined based on a previous proposal, grouping anatomically related channels (see Figure 2). Additionally, averages from the left PFC (LPFC), right PFC (RPFC), left medial PFC (LMPFC), and right medial PFC (RMPFC) were considered [12,36,37].

### 2.5. Data Analysis

Categorical variables were reported as frequencies and percentages, and the chi-square [χ^2^] or Fisher’s exact tests was applied as appropriate. The Shapiro–Wilk test was used to assess the normality of continuous variables, expressed as mean and standard deviation. Student’s *t*-test was used to compare group means for continuous variables when the normality assumption was met. When normality was violated, the non-parametric Mann–Whitney U test was applied to compare median values. Levene’s test was used to assess the homogeneity of variance for continuous individual variables. No imputation was performed for missing data; therefore, only data from participants who completed the endpoint assessment were analyzed. The effects of iCS on outcomes (MMSE, FAB, GDS-15) were analyzed using 2 × 2 repeated-measures mixed ANOVAs, with Group Assignment (iCS, control group) as the between-subjects factor and Time (baseline [T0], endpoint [T1]) as the within-subjects factor. The primary effects of interest were the Group × Time interactions. Post hoc pairwise comparisons with Bonferroni correction were conducted to compare groups (iCS vs. control group) at each time point (T0, T1) and within-group changes across time points (T0 vs. T1) for both iCS and control groups. ROI (four prefrontal cortex locations, as described previously), concerning fNIRS statistical analysis. The Greenhouse–Geisser correction was used when sphericity was violated. A significance level of 5% was set, with a two-tailed *p*-value < 0.05 was considered statistically significant. The effect size in ANOVA was assessed using partial eta squared (*ηp*^2^). Statistical analysis was performed using IBM SPSS Statistics, version 29.

## 3. Results

### 3.1. Sociodemographic and Clinical Characteristics

The sample for this study was selected from a randomized clinical trial of iCS for people with mild NCD (clinicaltrials.gov ID: NCT04693611). Recruitment took place from May to July 2021. Of the 40 potential participants assessed for eligibility, four participants were excluded for severe sensory impairments (*n* = 2), diagnosis of movement disorders (*n* = 2). Among the 36 included participants, 18 (50%) were assigned to the intervention group and 18 (50%) assigned to the control group. Table 2 shows the participants’ characteristics and assessment scores at baseline, as well as the results of between-group comparisons. No significant differences were found between the intervention and control groups regarding age, gender, educational level, marital status, household members, and type of social care institution attended. No significant differences were found between the intervention and control groups regarding clinical condition, or baseline mean scores in MMSE, FAB, and GDS-15. Thus, this equivalence ensures that any differences observed post-intervention are likely attributable to the intervention itself, rather than pre-existing group disparities.

Of the 18 participants in the control group, one (5.6%) did not complete the endpoint assessment due to death (Figure 1). For those participants who completed the endpoint assessment, no significant differences were found between the intervention and control groups regarding demographic variables, clinical condition, or baseline mean scores for the outcomes.

### 3.2. Results in Behavioral and Cognitive Dimensions

#### 3.2.1. Mini-Mental State Examination (MMSE)

ANOVA (Table 3) revealed a significant Group × Time interaction, F(1, 33) = 20.103, *p* < 0.001, *ηp*^2^ = 0.379, with participants in the iCS group significantly improving their MMSE scores at endpoint assessment (Bonferroni correction *p* < 0.001) and those in the control group maintaining them (*p* = 0.144). Intervention and control groups differed significantly at endpoint assessment (*p* = 0.003).

#### 3.2.2. Frontal Assessment Battery (FAB)

A significant Group × Time interaction was found for the FAB total score, F(1, 33) = 30.153, *p* < 0.001, *ηp*^2^ = 0.477 (Table 3). Participants in the control group had worse FAB scores at endpoint assessment (*p* = 0.018), while participants in the intervention group increased significantly their FAB scores (*p* < 0.001). The groups differed significantly in the endpoint evaluation (*p* = 0.001).

ANOVAs for FAB subtests revealed significant improvements in conceptualization (interaction between Group × Time F(1, 33) = 11.406, *p* = 0.002, *ηp*^2^ = 0.257; pairwise comparisons for the intervention group T0 vs. T1, *p* < 0.001), motor programming (interaction between Group × Time F(1, 33) = 6.482, p = 0.016, *ηp*^2^ = 0.103; pairwise comparisons for the intervention group T0 vs. T1, *p* = 0.040; intervention group vs. control group at T1, *p* = 0.002), sensitivity to interference (interaction between Group × Time F(1, 33) = 7.720, *p* = 0.009, *ηp*^2^ = 0.190; intervention group vs. control group at T1, *p* = 0.011), and environmental autonomy (interaction between Group × Time F(1, 33) = 4.653, *p* = 0.038, *ηp^2^* = 0.124; pairwise comparisons for control group T0 vs. T1, *p* < 0.001). No significant effect was found for mental flexibility (interaction between Group × Time F(1, 33) = 3.771, *p* = 0.061, *ηp*^2^ = 0.103, although significant differences were observed at the endpoint evaluation (*p* = 0.007) and pairwise comparisons for the control group T0 vs. T1, *p* = 0.005), and inhibitory control (interaction between Group × Time F(1, 33) = 0.027, *p* = 0.870, *ηp*^2^ = 0.001, although with differences significantly in pairwise comparisons for both groups T0 vs. T1 (*p* < 0.001)).

#### 3.2.3. Geriatric Depression Scale-15 (GDS-15)

ANOVA for GDS-15 revealed a significant Group × Time interaction, F(1, 33) = 18.945, *p* < 0.001, *ηp*^2^ = 0.365 (Table 3). This significant effect reflects a reduction in depression scores in the intervention group after iCS (pairwise comparisons for the intervention group T0 vs. T1, *p* < 0.001; intervention group vs. control group at T1, *p* = 0.012).

### 3.3. Results of Prefrontal Cortex Oxygenation in the Intervention Group

**Figure 3:**
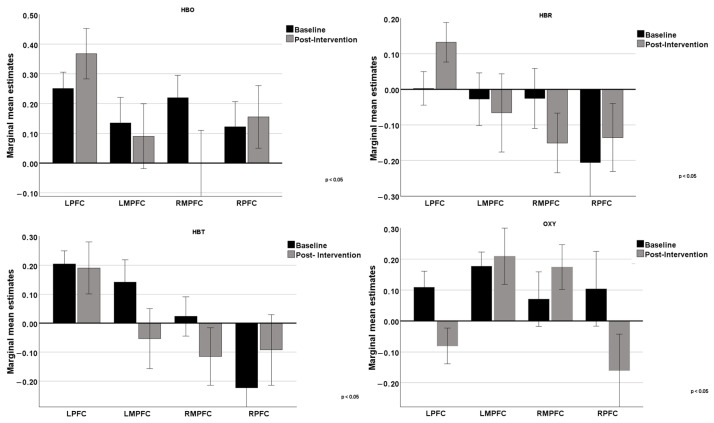
Comparison of the 4 regions of the prefrontal cortex (LPFC, LMPFC, RMPFC, and RPFC) at baseline and post-intervention on the 4 variables extracted in fNIRS.

As shown in Figure 3, a comparison was made between the baseline values and post-intervention values for cognitive stimulation of HBO, HBR, HBT, and OXY in the four regions of interest (LPFC, LMPFC, RMPFC, and RPFC), only for the iCS.

Regarding HBO, the results obtained suggest that cognitive intervention had different effects on various regions of the prefrontal cortex. The lateral areas (LPFC and RPFC) showed an increase in blood oxygenation, indicating potentially higher activation or functional efficiency in these regions post-intervention. The more medial areas (LMPFC and RMPFC) did not show significant changes, suggesting that the intervention may not have had a notable impact on these regions, or that the effects were not strong enough to be detected with the measures used. These results may reflect that the specific cognitive functions trained by the intervention are more associated with the lateral regions of the prefrontal cortex, while the medial regions were not as directly involved or affected by the training.

Regarding HBR, cognitive intervention impacted the functions of the lateral regions of the prefrontal cortex, which may indicate higher metabolic demand or reduced oxygen supply in these specific brain areas. On the other hand, the medial regions did not show significant changes, suggesting that the intervention had a lesser impact on these areas or that the effects were not adequately captured. The results for HBT highlighted a positive impact in the RPFC region, as evidenced by an increase in HBT concentration. The other regions showed decreases or no significant change, suggesting that the intervention may have more specific or localized effects. Results suggest that the RPFC region may be more sensitive or responsive to cognitive intervention. Regarding the OXY parameter, there was an increase in OXY difference post-intervention in the medial regions (LMPFC and RMPFC), suggesting improved oxygenation in these areas of the prefrontal cortex. In contrast, the LPFC and RPFC regions showed a decrease in OXY difference post-intervention, indicating a possible reduction in oxygenation variation in these areas of the prefrontal cortex. These results suggest that cognitive intervention had different effects on various regions of the prefrontal cortex, increasing oxygenation in some areas while decreasing it in others.

**Figure 4:**
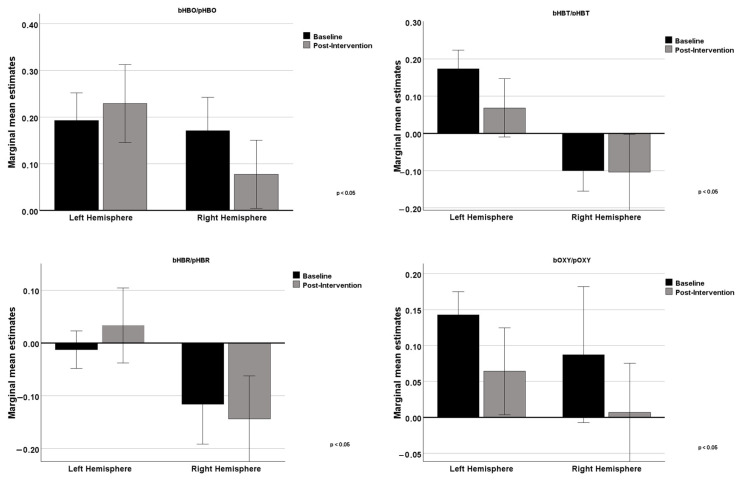
Comparison between baseline and post-intervention measurements in the left and right hemispheres for the 4 variables.

In Figure 4, we can analyze the graphs comparing the differences between the left hemisphere and the right hemisphere concerning the four variables. In the upper left panel, regarding HBO levels, baseline values were slightly higher in the left hemisphere compared to the right. After the intervention, HBO levels increased in the left hemisphere and slightly decreased in the right, indicating an asymmetric effect on cerebral oxygenation. HBR, represented in the upper right panel, shows negative baseline levels in both hemispheres. After the intervention, HBR levels increased in the left hemisphere (becoming positive) and further decreased in the right hemisphere, showing opposite effects between the hemispheres. In the lower left panel, HBT levels were positive in the left hemisphere and slightly negative in the right at baseline. After the intervention, HBT levels significantly decreased in the left hemisphere while remaining almost unchanged in the right. Finally, in the lower right panel, OXY levels were positive in both hemispheres at baseline, being higher in the left hemisphere. After the intervention, OXY levels significantly decreased in both hemispheres.

### 3.4. Adherence to Intervention

Adherence to iCS sessions was very high (Table 4). The mean attendance of participants was 23.8 sessions (out of 24 sessions), with 94.4% of participants attending more than 22 sessions and 88.9% of participants attended all sessions. The reasons for not attending the three sessions were hospitalization due to physical illness or a physical acute illness (66.7%), and medical appointments (33.3%).

### 3.5. Degree of Collaboration During the Intervention

We also obtained data on participants’ level of engagement throughout the intervention program. Participation was very high. Of the 432 iCS sessions (including all participants in the intervention group), the participants completed 429 (99.3%) of them. Participants appeared minimally engaged in three (0.7%) sessions, according to the qualitative judgment provided by the therapists at the end of each session.

## 4. Discussion

This single-blind RCT evaluated whether iCS in older adults with mild NCD positively impacts executive and other cognitive functions, functional abilities, and mood. Cognitive and behavioral instruments were administered to both the intervention and control groups. fNIRS was used to measure changes in oxygenated and deoxygenated hemoglobin before and after the 12-week intervention, providing insights into neurophysiological changes.

The intervention in this study specifically targeted cognitive domains including executive function, memory, and attention, through a structured intervention program. These included verbal fluency tasks, problem-solving exercises, and working memory tasks designed to stimulate neuroplasticity in the PFC. By engaging participants in these targeted activities, the iCS program aimed to enhance cognitive efficiency and thereby promote sustained activation of key neural regions associated with cognitive processing.

We found that behavioral and cognitive responses to the instruments depended on the timing and duration of the intervention (e.g., baseline vs. post-intervention assessments), suggesting that the intervention was beneficial. Participants in the intervention group showed significant improvements compared to the control group, supporting its effectiveness.

Our iCS intervention successfully improved performance in memory and executive functioning, as assessed by the MMSE and FAB, and reduced depressive symptoms, as measured by GDS-15. These findings reinforce previous studies highlighting the positive effects of cognitive stimulation on cognitive function and emotional well-being in older adults with mild NCD [39,40].

The reduction in depressive symptoms observed in this study is particularly relevant, given the high prevalence of mood disorders in older adults with mild NCD. Previous research has demonstrated that cognitive stimulation interventions can positively impact mood, likely through mechanisms related to increased social engagement, cognitive empowerment, and neuroplasticity [10,19]. Cognitive stimulation may help regulate affective responses by engaging prefrontal cortex regions associated with emotional processing and executive control [18]. Moreover, as indicated by the fNIRS data, the intervention was associated with increased activation in the lateral prefrontal cortex, a region implicated in mood regulation. This aligns with prior findings suggesting that cognitive interventions can modulate neural activity in ways that alleviate symptoms of depression in older adults with cognitive impairment [19].

Beyond cognitive improvements, neurophysiological changes were observed. An increase in HBO in the lateral regions of the prefrontal cortex (LPFC and RPFC) and changes in HBT suggest that cognitive tasks targeted in the intervention predominantly engaged the lateral prefrontal cortex, while medial regions were less affected. A significant increase in HBT in the RPFC indicates a potential enhancement in cognitive function or an increased metabolic demand due to the intervention [41,42]. However, it is important to note that fNIRS data were collected only from the intervention group, without a direct comparison to the control group. Therefore, it is not possible to conclusively attribute these changes in neural activity solely to the intervention. The lack of a control group in the neurophysiological measurements limits the causal interpretation of these findings, and results should be interpreted with caution.

These findings are consistent with previous research demonstrating the effects of cognitive training on specific regions of the prefrontal cortex [40]. However, as Figure 4 suggests, the intervention also contributed to an asymmetric effect on cerebral oxygenation levels, with an increase in the left hemisphere and a decrease in the right hemisphere. This asymmetry might reflect the nature of the cognitive activities emphasized in the intervention, which may have selectively engaged left-hemisphere-dominant processes such as verbal memory, language fluency, and rule-based executive control, particularly activating the left LPFC [43,44]. Nonetheless, this interpretation remains exploratory, as the current study was not specifically designed to test hypotheses regarding lateralization; alternative explanations, such as inter-individual variability, vascular asymmetries, or task habituation, cannot be ruled out. Therefore, conclusions regarding hemispheric asymmetry should be interpreted with caution and considered preliminary. The predominant fNIRS finding remains the increased activation in the lateral PFC following the intervention, which is plausibly associated with the observed cognitive improvements, particularly in executive functioning and memory. In line with prior studies [42,45], we observed that cognitive training interventions can modulate prefrontal cortex activity. However, due to the absence of fNIRS data from the control group, our findings should be interpreted as preliminary evidence of neural changes rather than definitive proof of intervention-specific effects. Future research should include neural activity measurements in both experimental and control conditions to strengthen causal conclusions.

Adherence to the intervention and the degree of collaboration were very high: 94% of the participants engaged in 23–24 sessions (average was 23.8 of 24 sessions attended). This strong adherence aligns with previous studies using similar iCS programs and supports the feasibility of individualized cognitive interventions in structured environments [21,22,46]. The one-to-one format appears to enhance participant engagement, particularly when implemented in controlled settings such as day centers.

These findings have clinical relevance. In particular, iCS was associated with improvements in global cognition in older adults with mild cognitive impairment. Significant improvements in mood were also observed following the intervention. Furthermore, fNIRS data indicated increased activation in the prefrontal cortex post-intervention. The use of individualized cognitive tasks appear to have enhanced the effectiveness of the intervention. Overall, the results support the use of non-pharmacological strategies to promote cognitive health in this population.

Furthermore, this is one of few studies to successfully integrate fNIRS to assess the neurophysiological impact of cognitive stimulation. The increased activation observed in the prefrontal cortex following the intervention highlights not only the functional responsiveness of this brain region to targeted cognitive tasks, but also supports the utility of fNIRS as a promising tool for monitoring treatment effects in clinical research. Although an asymmetric pattern of activation was observed—characterized by increased oxygenation in the left PFC and decreased activation in the right—this finding should be interpreted cautiously. Lateralization may be related to the verbal- and rule-based natures of the cognitive tasks, which are typically associated with left-hemisphere dominance. However, given the exploratory nature of this analysis and the lack of neurophysiological data from a control group, definitive conclusions cannot be drawn. Future research should more explicitly investigate lateralized brain responses to targeted cognitive domains.

Taken together, these findings reinforce the clinical value of personalized cognitive interventions and support the integration of neuroimaging methods such as fNIRS in future trials to enhance understanding of treatment mechanisms and optimize care strategies for individuals with mild NCD.

However, the study faced some limitations. The small sample size (*N* = 36) may have reduced the statistical power to detect smaller effects, which limits the generalizability of the findings. It also precluded subgroup analyses, such as comparisons between different NCD etiologies. Although group equivalence was achieved at baseline, no a priori power calculation was conducted, which would have strengthened the methodological rigor of the trial. We recommend that future studies include power analyses to ensure adequate statistical power. Furthermore, fNIRS data were collected only from the intervention group, without a control group for comparison, which limits causal interpretations regarding the observed neurophysiological effects. Thus, the observed PFC changes may partly reflect non-specific factors such as time effects or repeated exposure to the task, and should be interpreted with caution.

In addition, only data from the first and last intervention sessions were analyzed. While the observed changes were significant, the lack of intermediate session data limits our ability to explore the trajectory of cognitive and hemodynamic adaptations throughout the program. These methodological constraints should be considered when interpreting the findings. Nonetheless, they do not diminish the promising nature of the results. Future research should address these limitations by including larger and more diverse samples, full longitudinal fNIRS data, and neurophysiological assessments in both intervention and control groups.

While screening tools such as the MMSE were used in this study, more comprehensive cognitive assessments, such as the ADAS-Cog, might offer deeper insights into specific domains of cognitive improvement.

Other limitations of the study are related to the lack of follow-up of the participants after the intervention period.

Despite these limitations, the significant changes observed pre- and post-intervention support the therapeutic potential of the program and highlight the need for further research with a larger and more diverse sample.

## 5. Conclusions

This study provides compelling evidence that the iCS program used is an effective non-pharmacological intervention for improving executive functioning and reducing depressive symptoms in older adults with mild neurocognitive disorder. The observed improvements in cognition and mood underscore the dual benefit of iCS in supporting both cognitive health and emotional well-being in this vulnerable population. Furthermore, fNIRS provides valuable insight into the neurophysiological mechanisms underlying these effects, reinforcing the therapeutic potential of the program and highlighting the importance of integrating individualized cognitive interventions with neuroimaging methods in future research.

## Figures and Tables

**Table 1:** Basic structure of the iCS program adapted from Justo-Henriques [20].

Duration.	Contents	Materials and Activities
5 min	Start of session	Greeting Mood assessment Session agenda
10 min	Orientation	Temporal and physical orientation
25 min	Stimulation of cognitive domains	Scheduled main session activities (presented in digital format via Microsoft PowerPoint) covering six themes: - Portuguese language (identify missing letters in words, synonyms or associated words, alphabetize, memorize words (timed), develop a related theme); - Mathematics (calculations, memorize results or numbers (timed), simulate purchase transactions, sort numbers); - Daily activities (naming objects, timed memorization of objects, comparing utensils, categorizing (e.g., clothing, footwear, food, medication, technical aids, personal hygiene products, kitchen utensils, and rooms in a house)); - The past (identify, name, and associate images from the present and the past, such as modes of transportation, appliances, housing, media, professions, clothing, celebrities, politics, regional and local references, identify similarities and differences, memorize (timed) past historical images; - Fruit (identify fruits through images or riddles, memorize (timed) the fruits presented and their sequence, associate fruits with the harvest seasons, associate fruits with other products derived from them, associate images of fruits with words); - Sounds (associating sounds with images, memorizing (timed) sounds and sequences, associating musical themes with interpreters; identifying names of musical themes; associating musical themes with geographical areas, such as cities, regions, archipelagos; identifying and counting words in a musical theme).
5 min	End of session	Processing and debriefing Session evaluation Closing

Abbreviations: iCS = individual cognitive stimulation.

**Table 2:** Participants’ characteristics and baseline assessment scores between-group comparisons.

	Overall Sample (*N* = 36)	iCS Group (*n* = 18)	Control Group (*n* = 18)	*t*, χ^2^	Fisher Exact Test	*p*-Value	*d*, φ, *U*
Age in years, Mean (SD) [range]	75.3 (7.81) [65–90]	74.8 (7.76) [65–84]	75.8 (8.05) [65–90]	*t* = −0.38		0.719	*U* = 174.00
Gender (%)							
Male	11 (30.6)	6 (33.3)	5 (27.8)	χ^2^ = 0.13		0.717	φ = 0.06
Female	25 (69.4)	12 (66.7)	13 (72.2)				
Educational level (%)							
1 to 4 years	28 (77.8)	13 (72.2)	15 (83.3)		0.570		φ = 0.31
5 to 6 years	2 (5.6)	2 (11.1)	0 (0)				
7 to 11 years	1 (2.8)	1 (5.6)	0 (0)				
more 11 years	5 (13.9)	2 (11.1)	3 (16.7)				
Marital status (%)							
No partner	20 (55.6)	11 (61.1)	9 (50)	χ^2^ = 0.45		0.502	φ = 0.11
With partner	16 (44.4)	7 (38.9)	9 (50)				
Household members (%)							
Alone	13 (36.1)	7 (38.9)	6 (33.3)	χ^2^ = 0.17		0.920	φ = 0.07
Spouse	12 (33.3)	6 (33.3)	6 (33.3)				
With relatives	11 (30.6)	5 (27.8)	6 (33.3)				
Type of social care institution attended (%)							
Day care	12 (33.3)	7 (38.9)	5 (27.8)	χ^2^ = 0.50		0.480	φ = 0.12
None	24 (66.7)	11 (61.1)	13 (72.2)				
Clinical condition (%)							
mNCD_Alzheimer disease	16 (44.4)	9 (50)	7 (38.9)		0.738		φ = 0.25
mNCD_TBI	1 (2.8)	0 (0)	1 (5.6)				
mNCD_FTD	1 (2.8)	0 (0)	1 (5.6)				
None	18 (50)	9 (50)	9 (50)				
Outcome							
MMSE score, Mean (SD) [range]	26.86 (2.10) [23–30]	26.50 (2.26) [23–30]	27.22 (1.93) [24–30]	*t* = −1.03		0.309	*d* = −0.34
FAB score, Mean (SD) [range]	10.44 (2.72) [6–17]	10.39 (2.95) [6–17]	10.50 (2.55) [7–15]	*t* = −0.12		0.905	*d* = −0.04
GDS-15 score, Mean (SD) [range]	7.03 (3.17) [0–12]	6.89 (3.25) [1–12]	7.17 (3.17) [0–12]	*t* = −0.26		0.797	*d* = −0.09

Abbreviations: FAB = Frontal Assessment Battery; GDS-15 = Geriatric Depression Scale-15; iCS = individual Cognitive Stimulation; MMSE = Mini-Mental State Examination; mNCD = mild neurocognitive disorder.

**Table 3:** Results of repeated measures ANOVA for iCS vs. Control Group at Baseline (T0), and Endpoint (T1) Assessments.

	iCS (*n* = 18)		Control (*n* =17)		Group × Time			Pairwise Comparisons			
	T0 Mean (SD)	T1 Mean (SD)	T0 Mean (SD)	T1 Mean (SD)	*F*	*p*-Value	*η_p_* ^2^	T0	T1	iCS	Control
								iCS vs. Control	iCS vs Control	T0 vs. T1	T0 vs. T1
MMSE	26.50 (2.26)	28.56 (1.29)	27.24 (1.99)	26.59 (2.29)	20.103	<0.001	0.379	0.315	0.003	<0.001	0.144
FAB	10.39 (2.95)	12.94 (2.94)	10.65 (2.55)	9.41 (3.04)	30.153	<0.001	0.477	0.784	0.001	<0.001	0.018
FAB subtests:											
Conceptualization	0.94 (0.54)	1.83 (0.92)	1.53 (0.94)	1.47 (0.80)	11.406	0.002	0.257	0.030	0.224	<0.001	0.772
Mental flexibility	2.39 (0.92)	2.33 (0.77)	2.06 (0.66)	1.53 (0.87)	3.771	0.061	0.103				
Motor programming	1.56 (0.86)	2.06 (0.73)	1.65 (0.93)	1.29 (0.59)	6.482	0.016	0.164	0.764	0.002	0.040	0.151
Sensitivity to interference	1.78 (1.22)	2.11 (0.96)	2.00 (1.17)	1.24 (0.97)	7.720	0.009	0.190	0.586	0.011	0.235	0.011
Inhibitory control	0.83 (1.04)	1.83 (0.99)	0.47 (0.87)	1.41 (0.94)	0.027	0.870	0.001				
Environmental autonomy	2.89 (0.32)	2.78 (0.43)	2.94 (0.24)	2.47 (0.51)	4.653	0.038	0.124	0.594	0.063	0.346	<0.001
GDS-15	6.89 (3.25)	4.67 (2.85)	6.94 (3.11)	7.29 (3.02)	18.945	<0.001	0.365	0.962	0.012	<0.001	0.411

Note. Results of pairwise comparisons (Bonferroni correction) for baseline T0 and endpoint T1 assessments (iCS vs. control group). Results of pairwise comparisons (Bonferroni correction) for iCS and control groups (baseline T0 vs. endpoint T1 assessments). Abbreviations: FAB = Frontal Assessment Battery; GDS-15= Geriatric Depression Scale-15; iCS= individual cognitive stimulation; MMSE= Mini-Mental State Examination; T0 = baseline assessment; T1= endpoint assessment.

**Table 4:** Attendance statistics for the iCS sessions.

Attendance	*n* = 18
Sessions attended	
Mean (SD)	23.83 (0.51)
Number of sessions attended (%)	
From 0 to 20	0 (0.0)
From 21 to 22	1 (5.6)
From 23 to 24	17 (94.4)

Abbreviations: iCS = individual cognitive stimulation.

## Data Availability

The data that support the findings of this study are available from the corresponding author upon reasonable request. Trial Protocol: Clinicaltrials.gov ID: NCT04693611 (2020-12-31); Prefrontal Cortex Dynamics of the Elderly During a Cognitive Stimulation Programme.
